# *Alternanthera mosaic potexvirus*: Several Features, Properties, and Application

**DOI:** 10.1155/2018/1973705

**Published:** 2018-06-19

**Authors:** Ekaterina Donchenko, Ekaterina Trifonova, Nikolai Nikitin, Joseph Atabekov, Olga Karpova

**Affiliations:** Department of Virology, Lomonosov Moscow State University, Moscow 119234, Russia

## Abstract

*Alternanthera mosaic virus* (AltMV) is a typical member of the* Potexvirus* genus in its morphology and genome structure; still it exhibits a number of unique features. They allow this virus to be considered a promising object for biotechnology. Virions and virus-like particles (VLPs) of AltMV are stable in a wide range of conditions, including sera of laboratory animals. AltMV VLPs can assemble at various pH and ionic strengths. Furthermore, AltMV virions and VLPs demonstrate high immunogenicity, enhancing the immune response to the target antigen thus offering the possibility of being used as potential adjuvants. Recently, for the first time for plant viruses, we showed the structural difference between morphologically similar viral and virus-like particles on AltMV virions and VLPs. In this review, we discuss the features of AltMV virions, AltMV VLP assembly, and their structure and properties, as well as the characteristics of AltMV isolates, host plants, infection symptoms, AltMV isolation and purification, genome structure, viral proteins, and AltMV-based vectors.

## 1. Introduction

*Alternanthera mosaic virus *(AltMV) was first described in 1999 as “isolate 451/1” in the state of Queensland, Australia [1]. The new virus was isolated from* Alternanthera pungens* plants of the family* Amaranthaceae*. AltMV belongs to the genus* Potexvirus* and the family* Alphaflexiviridae*. The AltMV genome is a positive-sense single-stranded RNA 6604-6607 nt long depending on the isolates. AltMV virions represent flexible filamentous particles with helical symmetry made up of one type of coat protein (CP) subunits. The mean length of AltMV virions equals 554 nm [1], 536 nm [2], or 570 nm [3]. As reported by Mukhamedzhanova* et al*. [3], the virions are 13 nm in diameter. Recently AltMV virion diameter was corrected to 13.5 nm by means of cryoelectron microscopy [4].

Since plant viruses and virus-like particles (VLPs) are essentially safe for humans they seem promising for technological advances in a broad range of areas from microelectronics to developing candidate vaccines and adjuvants [5, 6]. AltMV virions and VLPs have a considerable number of advantages for successful application in biotechnology [4, 7–9].

## 2. AltMV Isolates and Their Distribution

Soon after AltMV had been discovered in Australia, other isolates were derived from plants in Europe [10, 11], USA [2, 12–16], Brazil [17], and Asia [18]. Nowadays AltMV is reported to be spread all over the world, and capable of infecting plants of various families including ornamental plants and crops [1, 19]. To date, complete nucleotide sequences are determined for the following AltMV isolates: AltMV-Ас (6604 nt long), AltMV-MU (6606 nt), and AltMV-PA (6607 nt). Four biologically active cDNA of “infectious clones” (3-1, 3-7, 4-1, and 4-7) were derived from AltMV-SP genome; the complete nucleotide sequences of these clones were established (6607 nt). The nucleotide sequences of the other isolates have been only partially determined [2, 14, 20].

The diversity of the obtained isolates both in terms of the host plants being the targets for virulence and geographical distribution implies the existence of phylogenetically diverged groups within the AltMV taxon [11, 20]. Based on amino acid sequences of RNA-dependent RNA polymerase (RdRp, replicase) and CP, Ivanov* et al*. [11] have distinguished 2 groups within the AltMV species: phlox-like isolates and portulaca-like isolates. In the study by Hammond and Reinsel [20] this differentiation was confirmed by means of amino acid sequence analysis of the three proteins which are products of the “triple gene block” (TGB). Apparently, Asian AltMV isolate should be regarded as a separate group [18].

AltMV-IT, AltMV-MU, AltMV-Port, AltMV-Po, AltMV-CIN, and AltMV-PLR (Table 1) were classified as portulaca-like isolates [20]. Ivanov* et al*. [11] indicated a close evolutionary relationship among the isolates of this group. The amino acid sequences of AltMV-IT, AltMV-MU, and AltMV-Po CPs differ on substitutions at two sites, methionine 106 for isoleucine and serine 185 for phenylalanine [11]. AltMV-Au, AltMV-PA, AltMV-NAN, and AltMV-SP were classified as phlox-like isolates. Belonging to different groups, the CPs of AltMV-MU and AltMV-PA are different by 12 amino acid residues situated mainly on the N-terminus of CP [11]. Nucleotide sequences determined for AltMV isolates obtained from* Scutellaria longifolia* [12],* Torenia* [17],* Angelonia angustifolia* [15], and* Thunbergia laurifolia* plants [21] are too short for detailed analysis, yet most likely they belong to portulaca-like type [20].

The reported distribution of isolates may be determined by the peculiarities of the host plant cultivation. Annual phlox, cineraria, angelonia, torenia, thunbergia, and portulaca are grown mostly in greenhouses as ornamental plants, while perennial phlox and nandina with vegetative reproduction are cultivated in open ground. Similar conditions of plant cultivation most likely account for portulaca-like AltMV detection in greenhouse plants; moreover, portulaca might have served as an infection source for this type of horticulture. Similarly, the spread of phlox-like AltMV obtained from plants cultivated in open ground was the result of cross-contamination from infected perennial phlox plants [20].

According to serological data as well as nucleotide and amino acid sequence similarity, AltMV is the closest relative of the* papaya mosaic virus* (PapMV) [1, 2, 20]. AltMV is easily confused with PapMV based on serological analysis or PCR analysis in case of an incorrect primer selection [2] which leads to AltMV being misinterpreted as PapMV [12, 22]. Hammond* et al*. [2] argued that AltMV is far more widespread than expected, especially in nurseries and greenhouses.

## 3. AltMV Host Plants and Infection Symptoms

AltMV has a broad host range and can infect plants from at least 31 taxonomic families including* Aizoaceae, Amaranthaceae, Apiaceae, Asteraceae, Brassicaceae, Caryophyllaceae, Chenopodiaceae, Cucurbitaceae, Fabaceae, Plantaginaceae, Polemoniaceae, * and* Solanaceae* [19]. The virus was detected in several ornamental species including* Portulaca grandiflora, Phlox stolonifera, Scutellaria* spp.,* Crossandra infundibuliformis, Angelonia angustifolia, Torenia* spp.,* Helichrysum* spp.,* Salvia splendens,* and* Zinnia elegans* [2, 12, 15, 17]. In addition to ornamentals, the systemically infected plants were found among various horticultural plants including* Solanum lycopersicum, Vicia faba, Helianthus annuus, Citrullus lanatus, Cucumis sativus, * and* Vigna unguiculata* [1]. The infected plants were mostly collected from commercial nurseries [2, 10, 12, 15, 17, 18, 21].

Upon being infected with AltMV all representatives of the* Amaranthaceae* family including* Alternanthera pungens* exhibited symptoms [1]. On the contrary, plants of the families* Caesalpiniaceae*,* Caricaceae*, and* Poaceae* are not susceptible to AltMV infection. As papaya* Carica papaya* is the main host for PapMV, its insusceptibility to AltMV confirms that these viruses belong to different species [1].

AltMV is known to manifest a wide range of symptoms including chlorotic spotting of various size, chlorosis, chlorotic local lesions, leaf distortion and curling, mosaic, mottle, necrotic spotting of various size, necrotic ringspots, rugosity, veinal necrosis, and interveinal yellowing (Table 2) [1, 2, 12, 15, 17].

AltMV infection symptoms were shown to depend not only on the host plant species, but also on the duration of infection, virus strain, and environmental factors. For example, symptoms being mostly pronounced in plants grown under high light levels and moderate temperature escaped notice in plants grown under low light levels and high temperature [2]. For AltMV-SP isolates derived from tobacco* Nicotiana benthamiana* the symptom severity was shown to correlate with sequence differences of the replicase (RdRp) and Triple Gene Block protein 1 (TGBp1) [23]. Similarly, in case of AltMV-Po isolate, the level of severity in symptom manifestation was demonstrated to go hand in hand with changes in amino acid sequence of CP [24].

The ability of AltMV to infect a wide host range leading to symptomless infection allows for cross-contamination among various species including the cultivated ones. Taking the aforementioned into account, AltMV may be more widespread than the literature suggests, particularly in nurseries [12, 20].

## 4. AltMV Propagation and Purification

Several methods were developed for AltMV isolation from a variety of host plants (Table 3). Geering and Thomas [1] followed the procedure previously described by Bancroft* et al*. [25]. The technique employed by Hammond* et al*. [2] was initially introduced for potexviruses and later adapted to potyviruses. In the studies by Mukhamedzhanova* et al*. [3] and Ivanov* et al*. [11] AltMV was isolated according to the protocol developed for another potexvirus, namely,* potato virus X* (PVX), with slight modifications. This technique was further substantially modified by Donchenko* et al*. [4].

Geering and Thomas [1] used* Chenopodium amaranticolor* as a host plant to propagate AltMV resulting in a yield of 23.4 mg of virus per 100 g of infected leaves. Even though the yield was relatively high, this host plant cannot be regarded as optimal for AltMV accumulation. Hammond* et al*. [2] managed to isolate AltMV from* Nicotiana benthamiana* with the yield of 8.6-12.5 mg of the virus per 100 g of green biomass while Mukhamedzhanova* et al*. [3] and Ivanov* et al*. [11] used* Portulaca grandiflora* as a host yielding 3.4 mg of virus per 100 g of infected leaves. Since* P. grandiflora* is hardly susceptible to infection with other viruses and* N. benthamiana* is a commonly used model plant, in the study by Donchenko* et al.* [4] portulaca and tobacco plants were selected as hosts [26]. In order to obtain purified AltMV, the infectious material was first propagated in* P. grandiflora* to prevent coinfection and later transmitted and accumulated in* N. benthamiana*. This allowed the yield to be substantially increased up to 20.0 mg and 57.3 mg of virus per 100 g of infected leaves in case of* P. grandiflora* and* N. benthamiana*, respectively [4].

## 5. Structure of AltMV Genome

The AltMV genome consists of a sole positive-sense single-stranded RNA having a cap at the 5' terminus and polyA sequence at the 3' terminus [1]. Nucleotide sequence analysis of AltMV-PA genome [2] revealed two untranslated regions (UTR) at the 5' (1-94 nt) and 3' termini (6481-6607 nt) respectively, as well as 5 open reading frames (ORF). The first ORF encountered at the 5' end of the genome is the longest (95-4720 nt) and is capable of encoding a 1540 amino acid (aa) long protein, namely, a viral replicase (RdRp). The next three ORFs presumably encode the three movement proteins referred to as Triple Gene Block Proteins: ORF2 (4704-5402 nt, encodes a 26 kDa 232 aa long protein); ORF3 (5356-5688 nt, encodes a 12 kDa 110 aa long protein); and ORF4 (5624-5815 nt, encodes a 7 kDa 63 aa long protein). The extreme 3′ end of the genome contains ORF5 (5858-6481 nt). Translation of the ORF5 produces a 22-23 kDa 207 aa residues long polypeptide being the CP. In addition, a comparatively short ORF6 was identified within all the presently known ORF5 of AltMV isolates [2, 11]. A sequence search through the Uniprot database failed to reveal any significant resemblance of the protein encoded by ORF6 to any known polypeptide. At present there is no evidence of ORF6 translation* in vivo* [2, 11].

The UTR of the AltMV genome is located in the vicinity of the ORF5 stop codon at the 3′ terminus. This 129 nt long genome region is highly conserved in AltMV isolates and the degree of nucleotide homology reaches 98% for some of them. This may be connected with the localization of the replicase recognition sites within this region [20]. Nevertheless, the secondary structure and the functions of the 3' UTR of the AltMV genome are yet to be determined. The same is also true for the 5' UTR as well as for putative regulatory elements of the AltMV genome. Furthermore, the presence of the conserved elements, namely, the octanucleotide and hexanucleotide motifs, suggests that their function in AltMV genome may be similar to that in the PVX one [2, 27]. Notably, no conserved polyadenylation signal similar to* bamboo mosaic virus* and PVX was detected in the 3′ UTR of AltMV [28].

## 6. *Alternanthera Mosaic Virus*-Based Vectors

To estimate the correlation of symptom severity and efficiency of cell-to-cell movement with various mutations in TGBp1 and AltMV replicase sequences Lim* et al*. [29] constructed a viral vector based on AltMV genome. The vector was further applied to outline the functions of AltMV TGBp3 [23] by means of fluorescent reporter proteins DsRed and GFP. Both works employed the same vector design with the reporter gene sequences being inserted between TGBp3 and CP genes under the control of the additional subgenomic (sg) promoter of AltMV CP [29].

A bipartite vector was also derived from AltMV-SP. Its first fragment contained AltMV replicase gene, the second one comprised TGB and CP gene of AltMV. In order to facilitate the cloning, the construction was divided into two parts: following plant tissue transformation with both fragments the complete AltMV genome was produced through recombination. The second component of the vector system was obtained in two versions. The vector was designed for target protein expression in plants and virus-induced gene silencing (VIGS) [23, 29]. Using the AltMV VIGS vector suppression of endogenous 4/1 protein of* N. benthamiana *expression and influence of Potato spindle tuber viroid movement in 4/1-silenced plants were demonstrated [30, 31]. Vectors AltMV-L-att and AltMV-P-att were created by insertion of the Gateway cloning cassette into the AltMV multiple cloning site (between the triple gene block and the CP gene) for protein expression and VIGS applications, respectively [32].

Several variants of the deconstructed viral AltMV-MU based vectors enabled heterologous proteins to be expressed in plants. TGB was deleted from the AltMV genome, while the target gene was placed under the control of either the AltMV additional sg promoter 1 (AltMV-single vector) or the two consecutive viral promoters sg promoter 1 and sg promoter 3 (AltMV-double vector) previously described in Lim* et al*. [23]. In comparison with AltMV-single, AltMV-double was demonstrated to produce higher target protein yield due to simultaneous functioning of the two sg promoters. AltMV CP and human granulocyte colony-stimulating factor were expressed as model proteins in the recent study [8]. Although an attempt was made to increase the protein accumulation level by using three sg promoters simultaneously, this approach failed to succeed [33].

## 7. AltMV Proteins

### 7.1. AltMV RNA-Dependent RNA Polymerase (RdRp)

Two predicted domains (the helicase and the polymerase ones) were identified in AltMV RdRp amino acid sequence by the BLAST algorithm [2]. Before helicase domain methyl transferase and 2-oxoglutarate-Fe(II) oxygenase domains are located [19]. Four variants of infectious clones derived from the AltMV-SP genome caused various symptoms in infected* N. benthamiana *plants and differed from each other in several amino acid substitutions in viral proteins including RdRp [23]. The clones were referred to as 3-1, 3-7, 4-1, and 4-7. None of them induced the infection symptoms similar to those of AltMV-SP isolate. Plant infection and two of the clones (3-7 and 4-7) lead to necrosis and eventual death, while in case of combining four of them milder symptoms were revealed. The replication rate increased at least 4 times at 15°С in all the clones. Plants inoculated with the mixture of 4-7 (‘severe') and 3-1 (‘mild') isolates developed symptoms similar to the ones caused by AltMV-SP, while the clone ratio was different at 25 and 15°С. The clones causing severe symptoms and high necrosis rate (3-7, 4-7) differed from those causing milder infection by several substitutions in the replicase amino acid sequence. Therefore, severe symptoms and higher necrosis rate were characteristic of P1110/R1121/K1255 replicase variant and milder symptoms, of R1110/K1121/R1255 variant. Notably, all the aforementioned amino acid substitutions were located in the polymerase domain of the protein [23].

The AltMV RdRp comprises 1540 aa with solely 68 varying among the isolates [20]. Although the difference by 45 amino acid substitutions between the RdRp of infectious clones 3-1 and 4-7 accounts for significant changes in replication efficiency [7, 29], only slight distinctions between the phlox and the portulaca isolates were detected through a phylogenetic tree analysis [20].

### 7.2. AltMV TGBp1

BLAST analysis performed for amino acid sequence of AltMV TGBp1 predicted the existence of N-terminal helicase domain [2].

Similarly to AltMV replicase, TGBp1 of AltMV-SP various clones manifest differences in their amino acid sequences. Part of the clones has leucine residue (TGBp1L88) and the other part has proline residue at 88 position (TGBp1P88) [29]. Solely TGBp1L88 is able to efficiently suppress posttranscriptional gene silencing in plants. Further investigation into the phenomenon revealed that the virus variant expressing TGBp1P88 has a lower replication rate in comparison with the variant expressing TGBp1L88. At the same time, both of the TGBp1 variants are capable of supporting the cell-to-cell movement although at different rates with TGBp1P88 slowing down the spread of infection. Notably, simultaneous expression of the two TGBp1 variants in plant cells reduces the antisilencing activity of the protein which implies the interaction between the two variants [29]. This interaction was confirmed using a yeast two-hybrid system. Subcellular localization of the TGBp1 variants by means of laser scanning confocal microscopy of* N. benthamiana* leaves indicated that TGBp1L88 is localized in the nuclear membrane and forms discrete aggregates in the nucleolus, while TGBp1P88 is localized in the nuclear periplasm [23]. Outside the nucleus TGBp1L88 was demonstrated to reside at the cell wall as small punctate aggregates, which suggests its association with plasmodesmata. On the contrary, TGBp1P88 was diffusely distributed throughout the cytoplasm. Since the helicase domain I of PVX TGBp1 has been previously reported to be required for* in vitro* oligomerization [34, 35], amino acid sequences alignment of AltMV TGBp1 and PVX TGBp1 was carried out in search for AltMV TGBp1 oligomerization sites. As a result, 7 conserved sequence motifs were identified in the helicase domain of AltMV TGBp1. The mutants carrying substitutions G31R and GK33/34RR in domain I of TGBp1 were unable to dimerize in the yeast two-hybrid system. The disrupted interaction was also observed* in vivo* in* N. benthamiana* plants by means of bimolecular fluorescence complementation. This argues for a crucial role of the protein domain I in the dimerization. As far as domains II and III are concerned, no mutations altered the dimerization process. The oligomerization of AltMV TGBp1 molecules is essential for silencing suppression [36]. Visualizing subcellular localization of AltMV TGBp1 variants both functional and defective in terms of oligomerization revealed the following pattern: AltMV TGBp1 variants capable of oligomerization were localized at the nucleolus or at the cell wall, while the mutant ones occupied the nucleoplasm instead of the nucleolus and were not detected in the vicinity of the cell wall [36]. These data suggest that TGBp1 oligomerization plays a key role both in cell-to-cell movement and silencing suppression.

AltMV TGBp1 is capable of selectively binding to several cellular proteins [37], namely, mitochondrial ATP synthase delta chain subunit, light-harvesting chlorophyll-protein complex I subunit A4, chlorophyll a/b binding protein, chloroplast IscA-like protein, and chloroplast *β*-ATPase. The latter was demonstrated to specifically bind solely to AltMV TGBp1L88 variant which is efficient silencing suppressor. At the same time, no interaction between the chloroplast *β*-ATPase and TGBp1P88 was detected. Since the virus-induced suppression of the protein expression induces severe symptoms in the host plant, the *β*-ATPase is considered to be involved in host plant immune response. Therefore, the interaction between the *β*-ATPase and TGBp1P88 appears to inhibit this process [37].

Similarly to PVX TGBp1, TGBp1 of AltMV is capable of interacting with one end of the virion thus activating RNA translation* in vitro* [3, 38].

### 7.3. AltMV TGBp2

To date, little is known about AltMV TGBp2 structure except for a transmembrane domain identified by BLAST amino acid sequence analysis [2]. Despite high sequence similarity with only six out of 110 amino acid residues varying among the isolates, portulaca-like AltMV-Po and AltMV-IT comprise a clade clearly distinct from the five phlox isolates, with the bootstrap value of 100% [20].

### 7.4. AltMV TGBp3

The three-dimensional structure of AltMV TGBp3 remains unresolved, and no specific domains have been revealed by BLAST search [2]. Up to now, two papers have addressed this issue [29, 39] and demonstrated AltMV TGBp3 to differ substantially from the homologous PVX one by subcellular as well as tissue localization patterns. In the infected leaves the fluorescently labeled TGBp3 was predominantly localized at the outer chloroplast membrane of mesophyll cells. Interestingly, TGBp3 overexpression induced chloroplast membrane vesiculation and veinal necrosis and contributed to the overall symptom severity. Deletion analysis indicated two amino acid residues (17V18L) of TGBp3 serving as the unique signal of AltMV TGBp3 localization in chloroplast membranes [29]. Moreover, TGBp3 is capable of directly interacting with the PsbO protein of the Photosystem II oxygen-evolving complex [39]. This interaction is governed by N-terminal region of TGBp3 from residue 16 to residue 20. The signal sequence required for AltMV TGBp3 chloroplast surface targeting is also localized within this region [29]. This may provide solid evidence for AltMV TGBp3 targeting chloroplast membrane through PsbO interaction, which in its turn is transported to chloroplasts from the cytoplasm where it is synthesized [39]. Thus, the efficiency of the interaction between PsbO and AltMV TRGp3 correlates with the severity of such symptoms as veinal necrosis and chloroplast membrane vesiculation. In case of impaired TGBp3 expression the virus lost the ability to enter the mesophyll cells and therefore cause systemic infection, which underlines the crucial role of TGBp3 in this process. Herewith, the defective virus demonstrated a comparatively limited ability to spread within epidermis with no systemic movement [29]. Visualizing the subcellular localization of AltMV RNA by means of fluorescence* in situ* hybridization indicated that the viral RNA as well as TGBp3 primarily accumulates near the surface of the chloroplast membrane. At the same time, the major amount of RNA was detected in mesophyll cells [29]. This drives to the conclusion that the AltMV replication occurs mostly in mesophyll cells, more specifically, at the outer chloroplast membrane. Interestingly, the presence of cellular TGBp2 exhibited no influence on AltMV TGBp3 subcellular localization as opposed to PVX TGBp3 [29, 40].

### 7.5. AltMV CP

AltMV coat protein (CP) is a 22-23-kDa protein comprising 207 aa. Together with AltMV replicase and TGBp1, AltMV CP determines symptom severity in host plants [24].

Both AltMV and PVX virions were found to be translationally activated. The AltMV genomic RNA is normally encapsidated and completely nontranslatable* in vitro*; however, translation can be activated through the phosphorylation of AltMV CP by protein kinase C or by TGBp1 binding to the viral particle [3].

AltMV CP was shown to assemble into stable extended polymers commonly referred to as VLPs* in vitro* under various conditions. Similarly to PapMV CP [41] AltMV CP formed extended VLPs* in vitro* in the absence of RNA at рН 4.0 and low ionic strength [3]. However, PapMV CP was incapable of forming RNA-free VLPs at pH 8.0, while AltMV CP formed particles morphologically resembling native virions under the same conditions [3]. In contrast to PapMV VLPs, AltMV ones were demonstrated to be highly stable under a wide range of conditions [3, 4]. According to their serological properties [3], virions and VLPs of AltMV are structurally different. Recent findings suggest that despite high morphological similarity, AltMV CPs possess a different fold in virions containing RNA and in RNA-free VLPs. By means of cryoelectron microscopy (CryoEM) the diameter of AltMV VLPs was measured to be 15.2 nm, thus exceeding that of AltMV virions (13.5 nm). Authors suggest that the absence RNA contributes significantly in increasing of VLPs central channel diameter (30 Å) versus virions (20 Å). CryoEM image processing demonstrated that VLPs possessed a larger number of CP subunits per turn (9.55) than AltMV virions (8.75) with the same pitch (35.7 Å) [4]. The authors hypothesize that, despite the similarity of AltMV virions and VLPs in the overall morphology when studied at low magnification, the folding and intersubunit interactions of AltMV CP differ in the presence and absence of RNA.

Tyulkina with colleagues [42] designed the hybrid viral vectors based on PVX genome and AltMV CP gene fused with sequences of influenza virus A M2e epitope. This vector was used for expression of chimeric AltMV CP in plant and VLP assembly. The authors considered this VLP as candidate vaccines [42]. Unlike PapMV there are no other works using AltMV virions or VLP as a platforms for epitopes presentation.

Both AltMV VLPs and virions demonstrated high stability under a wide range of conditions. It was shown that viral particles and VLPs do not change their morphology and size during incubation in distilled water, 0.15 M NaCl, and 0.01 M Tris-HCl, 0.15 M NaCl, and pH 7.5. Particularly worth mentioning is that AltMV virions and VLPs also remained stable after 1 hour incubation in mouse serum. Therefore, the absence of RNA in the VLP and the absence of RNA-protein interactions did not affect the stability of the protein helix of the AltMV VLPs under the selected conditions. [4]. Moreover, high immunostimulating properties resulting in significant enhancement of immune response to a model antigen in test animals were shown for both types of particles [43]. These data ensure the practical application of virions and VLPs of AltMV as an adjuvant platform for vaccine development. Both types of virus particles have numerous advantages such as assembly conditions and stability of the particles in comparison with the AltMV closest relative, namely, PapMV, that has been already applied most successfully in this field of research [44, 45].

## 8. Conclusion

*Alternanthera mosaic virus* (AltMV) is a representative of potexviruses with genome structure and virion morphology typical of the group. Additionally, AltMV has been proved to have various desirable properties in terms of its practical application. Moreover, the protocols for the virus particles production and purification have been elaborated establishing the foundation for their further application. Numerous viral vectors were derived from the AltMV genomes providing a perspective tool for target protein production in plants. Stability under a broad range of conditions as well as the immunostimulating properties make AltMV virions and virus-like particles a powerful tool for a plethora of biomedical applications.

## Figures and Tables

**Table 1:** AltMV isolates.

**Virus isolate**	**Accession no.**	**Authors**	**Original host**	**Origin of infected plants**
**Phlox-like isolates**				

AltMV-AU	AF080448	Geering and Thomas, 1999	*Alternanthera pungens*	Australia, QLD

AltMV-РА	AY863024	Hammond* et al.*, 2004, 2006a, b	*Phlox stolonifera*	USA, PA

AltMV-SP	AY850931	Hammond* et al.*, 2004, 2006a, b	*Phlox stolonifera* cv. Sherwood Purple	USA, MD

AltMV-BR	AY850928	Hammond* et al.*, 2004, 2006a, b	*Phlox stolonifera* cv. Blue Ridge	USA, MD

AltMV-NAN	GU126686	Tang *et al.*, 2010	*Nandina domestica*	USA, OK

AltMV-BW	JX457329	Hammond and Reinsel, 2015	*Phlox stolonifera *cv. Bruce's White	USA, MD

AltMV-LGB	JX457330	Hammond and Reinsel, 2015	*Phlox divaricata *cv. London Grove Blue	USA, MD

AltMV-PGL	JQ405265	Hammond and Reinsel, 2015	*Phlox carolina angusta*	USA

**Portulaca-like isolates**				

AltMV-IT	AY566288	Ciuffo and Turina, 2004	*Portulaca grandiflora*	Italy, Liguria, Albenga

AltMV-Po	AY850930	Hammond* et al.*, 2004, 2006a, b	*Portulaca grandiflora*	USA, MD

AltMV-MU	FJ822136	Ivanov *et al.*, 2011	*Portulaca grandiflora*	South-Eastern Europe

AltMV-Port	JQ405269	Hammond and Reinsel, 2015	*Portulaca grandiflora*	USA

AltMV-PLR	JQ405266	Hammond and Reinsel, 2015	*Phlox *hybrid (annual)	USA

AltMV-CIN	JQ405268	Hammond and Reinsel, 2015	*Pericallis *hybrid	USA

**Asian isolate**				

AltMV-Ас	LC107515	Iwabuchi *et al*., 2016	*Achyranthes bidentata*	Japan, Tokyo

**Unassigned isolates**				

AltMV Florida isolate	DQ393785	Baker *et al., *2006	*Portulaca *sp. *Scutellaria longifolia* *Crossandra infundibuliformis*	USA, FL

AltMV-Т	FJ232066, FJ232067	Duarte *et al.,* 2008	*Torenia* sp.	Brazil, São Paulo

AltMV angelonia isolate	EU679363	Lockhart and Daughtrey, 2008	*Angelonia angustifolia*	USA, NY

AltMV isolate G10-00982	JQ687034	Vitoreli *et al.*, 2011	*Thunbergia laurifolia*	USA, FL

**Table 2:** Diversity of AltMV host plants and infection symptoms.

**Family**	**Host**	**Symptoms**		**Authors**
		local	systemic	
*Aizoaceae*	*Tetragonia expansa*	necrotic ringspot	chlorosis, veinal necrosis, leaf curl	Hammond *et al.,* 2006b

*Amaranthaceae*	*Amaranthus caudatus*	necrotic local lesions	no infection	Hammond *et al.,* 2006b
	*Amaranthus tricolor*	chlorotic local lesions	mosaic	Geering and Thomas, 1999
	*Gomphrena celosiodes*	asymptomatic infection	mosaic	
	*Gomphrena globosa*	necrotic local lesions	asymptomatic infection	Geering and Thomas, 1999; Hammond *et al.,* 2006b
	*Alternanthera dentata*	necrotic local lesions	necrotic spotting, distortion	Hammond *et. al,* 2006b

*Apiaceae*	*Apium graveolens*, cv. Crisp Salad	necrotic local lesions	mosaic	Geering and Thomas, 1999

*Asteraceae*	*Aster novi-belgii*	no infection	no infection	Hammond *et al.,* 2006b
	*Dahlia variabilis*	no infection	no infection	
	*Helianthus annuus*	asymptomatic infection	no infection	
	*Sanvitalia procumbens*	no infection	mosaic, leaf curl, distortion	
	*Lactuca sativa,* cv. Black velvet	necrotic local lesions	no infection	Geering and Thomas, 1999
	*Zinnia elegans*	asymptomatic infection	mottle/ asymptomatic infection	Geering and Thomas, 1999/Hammond *et al.,* 2006b

*Brassicaceae*	*Brassica campestris *var.* pekinensis *cv. Lin White Spoon	asymptomatic infection	no infection	Geering and Thomas, 1999
	*Rhaphanus sativus *cv. French Breakfast	no infection	no infection	

*Caesalpiniaceae*	*Cassia floribunda*	no infection	no infection	Geering and Thomas, 1999
	*Cassia occidentalis*	no infection	no infection	

*Caricaceae*	*Carica papaya *cv. Richter Gold	no infection	no infection	Geering and Thomas, 1999

*Caryophyllaceae*	*Gypsophila repens*	asymptomatic infection	no infection	Hammond *et al.,* 2006b

*Chenopodiaceae*	*Chenopodium amaranticolor*	chlorotic local lesions	mosaic	Geering and Thomas, 1999
	*Chenopodium quinoa*	chlorotic local lesions/necrotic local lesions	interveinal yellowing/no infection	Geering and Thomas, 1999/Hammond *et al.,* 2006b
	*Spinacia oleracea*	chlorotic local lesions/no infection	mosaic/necrotic fleck, leaf curl	

*Cucurbitaceae*	*Citrullus lanatus *var. *Caffer *cv. Candy Red	asymptomatic infection	mosaic	Geering and Thomas, 1999
	*Cucumis sativus *cv. Green Gem	asymptomatic infection	asymptomatic infection	
	*Cucumis sativus* (two cultivars)	no infection	no infection	Hammond *et al.,* 2006b
	*Cucurbita pepo *cv. Green Buttons	asymptomatic infection	no infection	Geering and Thomas, 1999

*Fabaceae*	*Glycine max *cv. Bragg	no infection	no infection	Geering and Thomas, 1999
	*Phaseolus vulgaris *cv. Bountiful	no infection	no infection	Geering and Thomas, 1999/Hammond *et al.,* 2006b
	*Phaseolus vulgaris *cv Kerman	no infection	no infection	
	*Pisum sativum *cv. Greenfeast	no infection	no infection	
	*Trifolium pratense *cv. Montgomery	asymptomatic infection	no infection	Geering and Thomas, 1999
	*Vigna unguiculata *cv. Black-eye	asymptomatic infection/no infection	asymptomatic infection/no infection	Geering and Thomas, 1999/Hammond *et al.,* 2006b
	*Vicia faba*	asymptomatic infection/no infection	mosaic/chlorotic fleck	

*Papaveraceae*	*Papaver orientale*	no infection	no infection	Hammond *et al.,* 2006b

*Plantaginaceae*	*Plantago lanceolata*	asymptomatic infection	mosaic	Geering and Thomas, 1999

*Poaceae*	*Sorghum halapense *cv. Silk	no infection	no infection	Geering and Thomas, 1999
	*Zea mays *cv. Jubilee	no infection	no infection	

*Polemoniaceae*	*Phlox drummondii*	no infection	mild mottle	Hammond *et al.,* 2006b
	*Phlox stolonifera*	mottle	mottle	

*Solanaceae*	*Capsicum annuum *cv. Yolo Wonder	no infection	no infection	Geering and Thomas, 1999/ Hammond *et al.,* 2006b
	*Datura stramonium*	no infection	no infection	Geering and Thomas, 1999
	*Solanum lycopersicum *cv. Gross Lisse	asymptomatic infection/no infection	mottle/mild mottle, leaf curl	Geering and Thomas, 1999/ Hammond *et al.,* 2006b
	*Nicotiana benthamiana*	asymptomatic infection/chlorotic local lesions	mosaic, rugosity, epinasty	
	*Nicotiana clevelandii*	no infection	no infection/mild chlorosis	
	*Nicotiana edwardsonii*	no infection	no infection	Hammond *et al.,* 2006b
	*Nicotiana glutinosa*	no infection	no infection	Geering and Thomas, 1999/ Hammond *et al.,* 2006b
	*Nicotiana megalosiphon*	necrotic local lesions, necrotic ringspot	mosaic, necrotic fleck	Hammond *et al.,* 2006b
	*Nicotiana rustica*	no infection	no infection	
	*Nicotiana tabacum *cv. Turkish	no infection	no infection	Geering and Thomas, 1999/ Hammond *et al.,* 2006b
	*Nicotiana tabacum *cv. Xanthi	no infection	no infection	
	*Physalis floridana*	no infection	no infection	Geering and Thomas, 1999
	*Solanum melongena*	no infection	faint chlorotic spotting, rugosity	Hammond *et al.,* 2006b
	*Solanum tuberosum *cv. Sebago	no infection	no infection	Geering and Thomas, 1999

**Table 3:** Comparison of AltMV isolation procedures.

**Isolation and purification steps**	**Authors**			
	**Geering and Thomas, 1999**	**Hammond ** ***et al.*, 2006b**	**Mukhamedzhanova ** ***et al*., 2011; Ivanov *et al., *2011**	**Donchenko *et al.,* 2017**
**Yield (mg of virus/100 g of green plant biomass) and host plants used**	23.4 /*Chenopodium amaranticolor*/	8.6-12.4/*Nicotiana benthamiana*/	3.4 /*Portulaca grandiflora*/	20.0 /*Portulaca grandiflora*/ 57.3/*Nicotiana benthamiana*/

**Homogenization buffer**	0.02 М Sodium borate buffer, 0.5% Na_2_SO_3_, рН 8.2 (250 ml buffer per 150 g of leaves)	0.5 M K_2_H/KH_2_P0_4_, 0.5% Na_2_SO_3_, рH 8.4 (3-5 buffer volumes per weight of leaves)	0.3 М glycine-КОН, 1% Na_2_SO_3_, рН 7.5 (3 ml buffer per 1 g of leaves)	0.3 М glycine-КОН, 1% Na_2_SO_3_, рН 7.5 (3 ml buffer per 1 g of leaves)

**Virus enrichment from plant tissue**	centrifugation 0.5% Triton X-100	centrifugation 2% Triton X-100, 4% PEG *М*_r_ 8000, 2% NaCl	centrifugation 1% Triton X-100 5% PEG *М*_r_ 6000, 2% NaCl (Personal communication)	centrifugation 1% Triton X-100 Two stages of precipitation: (1) 5% PEG *М*_r_ 6000, 2% NaCl (2) 8% PEG *М*_r_ 6000

**Extraction of virus from pellets**	—	Extraction in 0.1 М Sodium borate buffer, 0.1 М KCl, рН 8.0 (BK buffer), 0.75-1.5 hours.	Extraction in 0.05 M Tris-HCl, 0.01 M EDTA, pH 8.0, (Personal communication)	Extraction in 0.05 М Tris-HCl, 0.01 М EDTA, рН 8.0, Two stages for 2-6 hours.

**Ultracentrifugation steps**	(1) Separation by 10-40% sucrose gradient (0.01 М Tris-HCl, 0.001 М EDTA, рН 8.0) (85 000 g, 4 hours) (2) Virus precipitation (85 000 g, 2.5 hours)	(1) Separation by 30% sucrose cushion (BK buffer) (85 600 g, 2.5 hours) (2) Separation by CsCl gradient, density 1.32 g/cm^3^ (139 000 g, 16-20 hours) (3) Dialysis against 0.5х BK buffer in 1 l (3 changes)	(1) Virus precipitation (100 000 g, 1.5 hours) (Personal communication) (2) Separation by 30% sucrose cushion (110 000 g, 2.5 hours) (Personal communication)	(1) Virus precipitation (111 000 g, 3 hours) (2) Separation by 30% sucrose cushion (extraction buffer) (111 000 g, 4 hours)

**Spectrophotometric analysis (** **E**_1 **cm**_ **,0.1**%_260 **nm**_**)**	2.84	2.5	2.84	2.84
